# Modular Breath Analyzer (MBA): Introduction of a Breath Analyzer Platform Based on an Innovative and Unique, Modular eNose Concept for Breath Diagnostics and Utilization of Calibration Transfer Methods in Breath Analysis Studies

**DOI:** 10.3390/molecules26123776

**Published:** 2021-06-21

**Authors:** Carsten Jaeschke, Marta Padilla, Johannes Glöckler, Inese Polaka, Martins Leja, Viktors Veliks, Jan Mitrovics, Marcis Leja, Boris Mizaikoff

**Affiliations:** 1 Institute of Analytical and Bioanalytical Chemistry, University of Ulm, Albert-Einstein-Allee 11, 89081 Ulm, Germany; carsten.jaeschke@gmx.de (C.J.); johannes.gloeckler@uni-ulm.de (J.G.); 2 JLM Innovation GmbH, Vor dem Kreuzberg 17, 72070 Tuebingen, Germany; marta.padilla@jlm-innovation.de (M.P.); jan.mitrovics@jlm-innovation.de (J.M.); 3 Institute of Clinical and Preventive Medicine, University of Latvia, LV-1079 Riga, Latvia; inese.polaka@gmail.com (I.P.); martinsleja11@gmail.com (M.L.); viktors.veliks@lu.lv (V.V.); marcis.leja@lu.lv (M.L.)

**Keywords:** breath analysis, MOX sensors, low sensing chamber volume, calibration transfer, standard samples, piecewise direct standardization, correlation alignment, breath sampling, eNose, pattern recognition

## Abstract

Exhaled breath analysis for early disease detection may provide a convenient method for painless and non-invasive diagnosis. In this work, a novel, compact and easy-to-use breath analyzer platform with a modular sensing chamber and direct breath sampling unit is presented. The developed analyzer system comprises a compact, low volume, temperature-controlled sensing chamber in three modules that can host any type of resistive gas sensor arrays. Furthermore, in this study three modular breath analyzers are explicitly tested for reproducibility in a real-life breath analysis experiment with several calibration transfer (CT) techniques using transfer samples from the experiment. The experiment consists of classifying breath samples from 15 subjects before and after eating a specific meal using three instruments. We investigate the possibility to transfer calibration models across instruments using transfer samples from the experiment under study, since representative samples of human breath at some conditions are difficult to simulate in a laboratory. For example, exhaled breath from subjects suffering from a disease for which the biomarkers are mostly unknown. Results show that many transfer samples of all the classes under study (in our case meal/no meal) are needed, although some CT methods present reasonably good results with only one class.

## 1. Introduction

The importance of exhaled breath gas analysis is increasing in medical diagnostics for early disease detection and therapy progress monitoring over the last decades [1,2,3,4]. Exhaled human breath is composed of nitrogen, oxygen, carbon dioxide, water vapor, inert gases and trace amounts of volatile organic compounds (VOCs) [5]. The ancient physician—Hippocrates (460–370 BC)—noticed that the exhaled breath of an ill patient differs from a healthy one and described fetor oris and fetor hepaticus in his essay on breath aroma and disease. In 1971 modern breath analysis started with the experiments of Pauling et al. [6], he showed that human breath contains several hundred different VOCs in low concentrations. Pauling’s observation was confirmed by subsequent studies from other research groups. Phillips et al. approved Pauling’s statement and confirmed that exhaled human breath contains more than a thousand different VOCs in low concentrations [7,8]. Furthermore, they observed, by gas chromatography coupled with mass spectrometry, 3481 different VOCs in the breath of 50 healthy humans. On average each human has approximately 204 VOCs in their breath. Moreover, it was observed that only 27 VOCs were equal in the breath of the 50 healthy humans examined. This makes breath analysis a difficult task because there is only a small common core of VOCs in all humans. These VOCs are probably produced by metabolic pathways common to most humans [8]. Moreover, these VOCs can be from exogenous and endogenous origin [9]. Exogenous VOCs are inhaled or absorbed as contaminants via breath, skin or ingestion [10] while endogenous VOCs are produced in the body via the metabolism [3]. The identification of these VOCs is very important and forms the focus of research, as they act or can act as important “markers” for the early detection of a disease [11]. The identification of breath markers should be qualitative and quantitative to distinguish between a diseased group and a healthy one. The differences in the VOCs content between these two groups must be large enough to reach clinical relevance. In the last 30 years, many of these molecules have been identified and correlated to different diseases. The basis of the emission of VOCs is cell biology. Tumor growth causes metabolic changes which are linked to the production of specific volatile compounds [12,13,14,15]. Cancer-related blood chemistry changes lead to changes in breath by exchange through the lung [16]. Therefore, some VOCs can be used as cancer markers in exhaled breath [3].

Currently, the gold standard for detecting VOCs in exhaled air is gas chromatography coupled to mass spectrometry (GC-MS) [1,17,18,19,20,21,22,23,24,25,26]. Beside GC-MS, other analytical instrumentations are used like proton-transfer reaction-mass spectrometry (PTR-MS) and ion mobility spectrometry (IMS). These techniques enable separation, identification and quantification of the different VOCs in the exhaled breath gas. The main disadvantages of the analytical instrumentation are the need of high skilled operating personnel, being time-consuming (except for IMS) and the high costs.

To reduce costs, chemical sensors integrated into electronic noses (eNoses) for breath analysis in medical point-of-care diagnosis have become an emerging field. Many research groups are pushing forward the frontier of non-invasive, rapid, portable and potentially low cost medical diagnosis tests for different diseases [3,27]. Electronic noses in breath gas analysis are still a noticeably young research field. Different research laboratories use different internal standardized methods for the breath sample collection, but there is no globally accepted standard procedure. The common procedures are total or alveolar breath gas sampling. In total breath sampling, the complete breath is collected including dead space air, and in the alveolar breath gas sampling only the end-tidal, alveolar part of breath is collected [1,28,29]. The method of total breath gas sampling is simple but has a big disadvantage because of the dilution with the dead space air [1]. In comparison, alveolar sampling reduces the concentration of contaminants [1,29].

Advantages and limitations of eNose sensor techniques are associated with different parameters like specificity, response and recovery time, detection range, sensitivity, operating temperature, temperature as well as humidity effect on sensing technique, portability, cost and complexity of measuring circuitry.

In the review by Röck et al., a list of commercially available eNoses is published [30]. Several preliminary studies were conducted with those commercially available eNose systems like Cyranose 320 [31,32,33,34,35,36], LibraNose [37] and DiagNose [38] using offline sampling with Tedlar or Mylar bags. Other studies with chemical sensors, surface acoustic wave (SAW) sensors [39], metal oxide semiconductor (MOX) sensors [40], colorimetric sensors [41], quartz microbalance (QMB) sensors [42,43], MOX-SAW sensors [44] and trichloro-(phenethyl)silane-silicon nanowire-field effect transistor (TPS-SiNW FET) sensors [45] were carried out. Furthermore, studies were conducted in which an eNose and additionally gas chromatography coupled to mass spectroscopy were used. Those studies showed that different organic functionalized gold nanoparticle (GNP) sensors are suitable to different diseases [46,47,48,49,50,51]. Amal et al. investigated the detection of gastric cancer utilizing GNP sensors with offline sampling and demonstrated that the sensor technology determines that the breath of cancer patients is different from healthy ones. [49,50].

Over recent years, the field of breath analysis with MOX-based eNose systems is continuously progressing. Different studies of cancer detection via breath gas analysis with eNoses based on MOX sensors have been conducted by Yu et al. [40], Wang, D. et al. [44] and Wang, X.R. et al. [52] and some of those studies also involved other sensor technologies in combination with a MOX sensor array. De Vries et al. integrated an eNose sensing array into an existing diagnostic spirometer. This system is based on five identical commercially available MOX sensor arrays out of four MOX sensors [53].

Special attention shall be given to the experiment design, most of the published studies on breath analysis by an eNose were conducted with one device. For larger scale studies, given the difficulties of obtaining breath measurements from patients with specific conditions, it would be desirable to extend the study to more devices and more places (like different hospitals or recruitment centers). However, sensor to sensor variability, time degradation (drift), cross-sensitivities to background and environmental conditions, etc., causes data models (calibration models) built for one instrument at a given time and place to not be valid for measurements collected by another instrument, or the same instrument in another place or later in time. In other words, the data calibration models degrade when taking measurements under conditions other than those under which the calibration model was created. The effort in building a calibration model is costly and time consuming, and therefore limits the use of eNoses in many applications such as breath analysis.

To reduce the impact of these limitations, data processing techniques exist that help in reducing the effort of full calibrations by transferring information from the main calibration model (built in a so-called master instrument) to be applied to new measurements obtained under different conditions (from so-called slave instruments). These techniques are called calibration transfer (CT) or instrument standardization in chemometrics, and transfer learning tools in machine learning. CT methods have been largely applied in NIR spectrometry and also in eNoses [54,55,56,57]. The objective of a calibration transfer method is to perform its task using as few transfer samples (samples to link instruments) as possible. In this way, the costs of calibration of individual (slave) instruments are reduced to a few measurements of transfer samples, instead of a whole large set for proper calibration.

Calibration transfer methods can be grouped according to different criteria. For example, the following approach [58]: (i) no standardization (feature selection, calibration model extension (CME) by including samples from multiples instruments, special pretreatments like orthogonal signal correction (OSC) [59]); (ii) adjusting the output of the calibration model to be used by other instruments, such as the simple univariate slope and bias correction (SBC) [60]; (iii) transforming measurements from slave instruments so that they resemble measurements from the master instrument using direct standardization (DS) and piecewise direct standardization (PDS) [61,62] and (iv) removing differences between instruments that are orthogonal to the calibration model [63]. Indeed, PDS has shown good results and is considered by many to be a reference for novel techniques [64,65,66,67,68,69].

Other classification of calibration transfer techniques can be made with regard to the domain of the transfer and the transfer samples. For the first one, we can have (a) transfer to the master space or the slave space, such as DS and PDS, (b) transfer to a common subspace such as OSC. Additionally, for the latter, CT methods regarding transfer samples; (a) methods that need transfer samples such as DS, PDS, OSC [70], Shenk’s algorithm [71], spectral space transformation (SST) [72] and canonical correlation analysis (CCA) [73] (b) and methods that do not need transfer samples, such as methods from the IR spectra field: multiplicative signal correction (MSC) [66], finite impulse response (FIR) [68,69], stacked partial least-squares (SPLS) [67] and from the machine learning field, like transfer component analysis (TCA) [74] and transfer sample-based coupled task learning (TCTL) [54] and others that have been applied to eNoses [75]. Reviews and discussions can be found in literature about the use of standard samples [76], about the techniques based on orthogonal projections [77,78] and reviews of different techniques [58,65,79].

An important matter in calibration techniques that need transfer samples is the selection of these transfer samples. Such samples must be representative of the samples under study and the respective instruments, keeping other variables such that they can be linked between the instruments. The required transfer samples would be those measured at the same conditions by different instruments, for example, by having the instruments measuring the same sample at the same time; we call them standard samples. When standard samples are not available, we can use reference (or nonstandard) samples, which are measurements made under exact or similar conditions in all master and slave instruments.

In addition, the fact that transfer samples must be representative of the samples under study may be a limitation in out-of-lab applications, such as breath analysis, because representative samples of given cases might be difficult to obtain. In an ideal situation, samples representative of the cases under study can be artificially made, such that measurements of these samples can be made in a laboratory under controlled conditions and thus be used as transfer samples. This way, all instruments would be referred to the same general samples under specified conditions. However, it is difficult to create synthetic samples representative of complex samples such as human breath samples, especially for patients suffering diseases for which the exhaled VOCs pattern is poorly known or even unknown and which is affected by numerous variables. Therefore, the use of on-site measured samples as transfer samples may be helpful, or alternatively hard-to-obtain sample classes could be excluded from the transfer sample set. In summary, for practical reasons we wonder whether on-site measured sample measurements can be used as transfer samples and whether the quality of the calibration transfer would decrease much if one sample class (necessary for the calibration model) is excluded from the transfer sample set. We can find an example in the literature where breath samples from electronic noses were used, although case breath samples were artificially made by mixing control breath samples with chemicals [57].

In this work, we explore the performance of several CT techniques in a real-life breath analysis study using our recently published [80,81] sensing array and three instruments. The experiment consists of the discrimination of breath samples from people before and after eating a specified meal. The performance of the CT methods is evaluated in regard to the number and type of transfer samples (standard or nonstandard) and class membership (transfer samples belonging to one class or both classes meal/no meal), to explore the possibility of using transfer samples from the on-site experiment in the CT methods.

**Figure 1:**
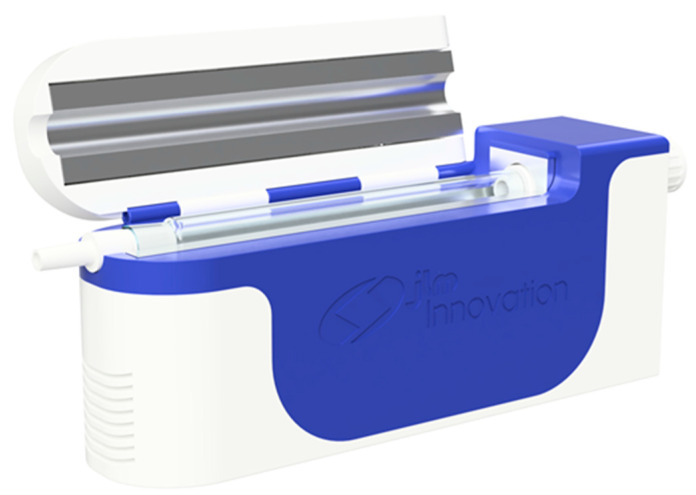
Modular breath analyzer (MBA) platform based on chemoresistive sensors for laboratory and clinical use; external view with cap opened to exchange the glass sampling tube.

This study is mainly motivated by the difficulty of calibration of eNoses for breath analysis applications, the differences between instruments, the frequent recalibrations needed due to aging and drift and the environmental and other different conditions present in different hospitals which prevents obtaining a unified dataset for deep statistical studies. Another goal in this work, is to present our modular breath analyzer (MBA) platform (shown in Figure 1), a new updated version of our modular eNose [80,81,82] specifically designed for breath analysis. The previous version of the modular eNose concept was recently presented [80,81,82]. It consisted of a new eNose platform based on a novel modular sensing chamber, where different kinds of chemoresistive sensors can be combined [80,81,82]. In one of these works, we combined analog and digital commercial surface mount devices (SMD) MOX gas sensors and checked its potential in an experiment aiming to detect VOCs under a high humidity background in a future application [82]. The other experiment consisted of testing the instrument for on-line monitoring under dry and moderate humid conditions with six concentrations of two VOCs of interest [80]. The MBA is based on the previously presented innovative iLovEnose concept of a modular eNose system [80,81] and incorporates a direct alveolar breath sampling system, which is explicitly used in the breath analysis experiment.

The manuscript is organized as follows. Section 2 describes our updated MBA instrument and details both experiments: the breath analysis study. Section 3 explains the analysis methods and followed methodology. Section 4 shows and discusses the results and finally Section 5 derives some conclusions.

## 2. Material

### 2.1. Device Description

**Figure 2:**
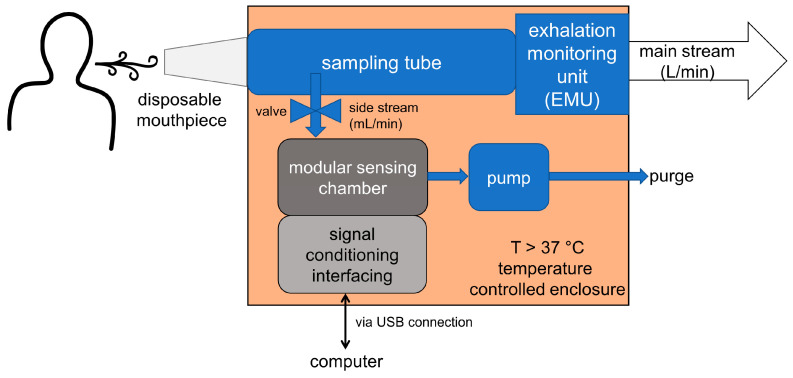
Schematic drawing of the modular breath analyzer platform for laboratory and clinical use showing the linkage of the individual units. Fluidic units are drawn in blue (sampling and exhalation monitoring unit) and dark gray (modular sensing chamber).

The presented novel modular breath analyzer (MBA) platform is developed for laboratory and clinical use. The internal components of the MBA platform are shown in Supplementary Materials, Figure S1, while its basic arrangement and the connection of the individual units is shown with a schematic drawing of the MBA platform in Figure 2. It contains a direct breath sampling unit and three modules able to host different types of sensors and technologies. The MBA platform contains three main units: (i) a sampling unit with an internal exhalation monitoring unit (EMU), (ii) a temperature control unit and (iii) a modular sensing chamber unit. The sampling unit is especially designed for breath analysis, based on the buffered-end-tidal (BET) sampling process [83]. Our presented system weights about 2.1 kg and has the dimensions 280 mm × 118 mm × 75 mm.

The two fluidic units—sampling unit as well as sensing unit—which are described below are integrated into a temperature-controlled aluminum body to ensure that all fluidic paths within the MBA platform are kept above body temperature to avoid temperature effects on the sensors to avoid influences towards the composition of the exhaled breath gas. Without thermostatic control the temperature within the fluidic paths will drop below body temperature and thus vapor will condense and trap water soluble volatile organic compounds (VOCs). For this reason, the temperature inside the sensing chamber of the presented modular breath analyzer platform is 45 °C ± 1 °C.

#### 2.1.1. Buffered-End-Tidal (BET) Sampling and Exhalation Monitoring Unit (EMU)

During an exhalation into the device the volume of the sampling tube is exchanged several times. After the exhalation process has finished, the last 38 mL of the exhaled breath are buffered within the tube and remain there until the sample is transferred into the sensing chamber by a controlled pumping process. The buffered volume of approx. 30 mL allows the system to transfer several times the volume inside the sensor chamber, which can be set by the software. The internal exhalation monitoring unit is coupled to the sampling tube to operate the pump directly when the exhalation stops to draw selected parts of the alveolar air inside the sensor chamber. Sensors within the EMU allow real-time monitoring of the full exhalation process. EMU parameters (like pressure, temperature, humidity) are recorded during the exhalation process of the volunteer or patient to ensure proper repeatable sampling and enable capturing relevant parts of the exhaled breath, which are different portions of the pulmonary volume.

For a reliable breath analyzer platform, it is crucial that the EMU is not only recognizing the end of the exhalation, it is also important to ensure that the volume of the exhaled breath and the profile is within a certain variance. Direct feedback to the patient and the study nurses may help to improve the sampling process. This enables capturing relevant parts of the exhaled breath and allows a more accurate transfer of sample to sensors as well as selective sampling of different portions of the pulmonary volume. To avoid cross-infection between the patients the glass sampling tube can be exchanged and cleaned by sterilization. The disposable mouthpiece is exchanged for every volunteer or patient.

#### 2.1.2. Modular Sensing Chamber Unit

**Figure 3:**
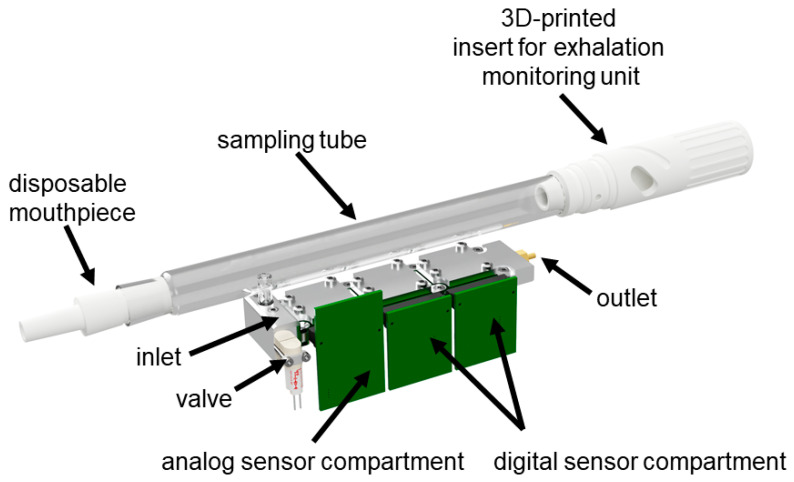
Sensing chamber with three individual compartments, shown here as a combination of analog and two digital metal oxide semiconductor (MOX) sensor compartments and the connection of the sampling tube to the valve-controlled sensing array inlet.

To utilize the buffered end-tidal breath sampling method, we designed a specific valve-controlled inlet for our recently published sensing chamber [80,81,82] to be able to plug the exchangeable glass sampling tube to the sensing chamber of our eNose system. The current sensing array setup consists of three compartments: one with 8 analog and two with 10 digital sensors each. The low volume of the sensing unit (less than 3 mL) ensures that the volume of the sensor chamber could be flushed several times with the BET air from the sampling tube. The sensing unit consists of a modular sensor array that contains three exchangeable sensor modules with a valve-controlled inlet connected to the sampling tube and one outlet (can be seen in Figure 3). The exchangeable modular design of the sensor unit allows the MBA to host three modules containing sensors of different types, but those three modules can also contain the same type of sensors, which is useful in the study of sensor chip variability.

The current setup contains many of the most relevant analog and digital surface mount devices (SMD) sensors on the market. A list of all integrated sensors, number of obtained signals from each sensor and used heater/supply voltages are summarized in Tables S1 and S2, see Supplementary Materials. The concept of the sensing unit follows a modular structure to allow an easy and simple exchange of the sensors [80].

The cleaning of the sensor chambers is done by a two-step process. First, the pump and the valve shown in Figure 2 and Figure 3 are used to generate a low vacuum for a few seconds to remove the breath gas out of the sensing chamber, and then ambient air is driven through the sensing chamber. The cleaning cycle is programmed in the firmware of the MBA, and it is started after a successful and a cancelled measurement.

### 2.2. Experiment–Pilot Study Description

**Figure 4:**
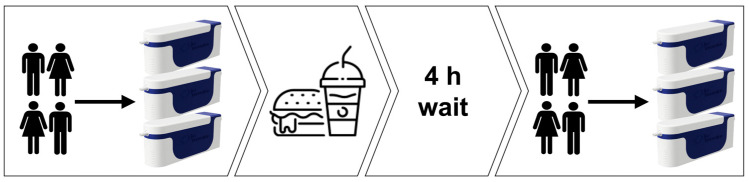
General scheme of collecting the breath samples for the pilot study.

A group of 15 generally healthy 17–18 year old individuals were recruited for an experimental study using three modular breath analyzer prototypes, the first breath sample was obtained following a 12 h fasting period with all three MBA devices; then the participants were given a standardized meal and invited for a follow-up (second) breath sample 4 h thereafter. The test-meal was a hamburger with 0.5 L water. To avoid potential contaminants, on the day of testing the recruited study participants did not use mouthwash, chewing gum, furthermore they did not perform excessive physical activity, did not smoke or consume alcohol for 24 h before the breath test. The same restrictions were applied to the 4-h pause between two measurements. This experimental routine was repeated three times, each measurement day was one week apart from the previous one. The general scheme of the breath sample collection (measurement day) of the pilot study can be seen in Figure 4. Signed consent was obtained from all recruited study participants. For study participants under 18 years of age the parents or legal guardians signed this consent.

In this study, the firmware of the used MBA devices was set to 10 s for baseline acquisition and 20 s for breath acquisition.

## 3. Methods

### 3.1. Calibration Transfer Algorithms

In this work, we compare three methods for calibration transfer based on different principles: Correlation alignment (CORAL) [84], partial least squares-based calibration transfer (PLSCT) [85], direct standardization (DS) and piecewise direct standardization (PDS) [61,86] and a partial least squares discriminant analysis-based method (PLSDA) [81].

DS, PDS and PLSCT are based on adjusting the slave features to the master by using a set of labeled transfer samples measured in both the master and the slave instruments. These samples can be standard samples, i.e., samples related to both instruments, such as same samples measured at the same time, samples measured at the exact same conditions, etc., or non-standard samples if they are labeled but not related as the standard samples. In turn, CORAL is a simple method that transforms data from the master to the slave space using their covariance structure without the need of labelled samples. Finally, PLSDA finds a common master-slave space by removing components using unlabeled transfer samples, only the information about their membership to the master or the slave instrument is used.

Direct standardization (DS) and piecewise direct standardization (PDS) methods—DS and PDS [61,86,87] methods were created in the field of NIR spectrometry to correct the slave spectra by computing a transfer matrix. This transfer matrix is obtained by relating the master spectra to the slave’s spectra by using a small number of labeled transfer samples. The PDS method is in fact an extension of DS by which each wavelength (variable) at the master spectra is related to a sliding window of fixed size in the slave spectra. PDS can deal with having a larger number of variables than samples [64].

DS and PDS are widely used methods which have provided good results in laboratory experiments using a relatively small number of samples [56,79] and are typically employed as a reference for other novel techniques [64].

DS assumes a linear relationship between master and slave instruments such that:

X^m^_ct_ = X^s^_ct_ B (1)

where B is the transformation matrix and X^m^_ct_ and X^s^_ct_ are the data measured from the transfer samples at the master and slave instrument, respectively. Therefore, B can be estimated by

B = (X^s^_ct_)^+^ X^m^_ct_ (2)

where (X^s^_ct_)^+^ is the pseudo inverse of X^s^_ct_. The new samples from the slave instrument X^s^ can be projected onto the master instrument X^m^:

X^m^ = X^s^ B (3)

In turn, PDS creates local PLS models relating the master instrument j-th variable to a sliding window of size w centered at the j-th variable in the slave instrument. The resulting transformation matrix B_PDS_ has a diagonal structure:

B_PDS_ = diag(b^1T^, b^2T^, …,) (4)

where k is the number of variables on both instruments. Finally, X^s^ can be projected on the master instrument by:

X^mT^ = X^sT^ B_PDS_ (5)

Partial least squares discriminant analysis-based calibration transfer (PLSDA)—the PLSDA-based method builds a PLSDA model relating transfer unlabeled samples from both master and slave instruments with a dummy vector containing their membership (master or slave) label. Furthermore, the predicted data from this model is removed from the original data set.

If W and P are notations for the resulting PLSDA weight and latent variable matrices, respectively, X^m^ and X^s^ denote the original data from the master and the slave instrument, respectively, and X^m^’ and X^s^’ denote the transformed data from the master and the slave instrument, respectively:

X^m^’ = X^om^ − X^om^ W ((P)^T^ W)^−1^ P (6)

X^s^’ = X^os^ − X^os^ W ((P)^T^ W)^−1^ P (7)

The number of components or latent variables (LVs) to be removed must be selected. Finally, a classification/regression method can be built on the X^m^’ and be used to predict X^s^’.

Partial least squares-based calibration transfer (PLSCT)—In PLSCT [85], a PLS model is built in the master instrument and a subset of samples are used to relate master and slave instruments. This operation is made in the PLS low dimensional space between projected spectra from transfer samples in both master and slave instrument.

If X^m^ and Y^m^ are the calibration set data and label matrices in the master instrument, respectively, W^m^, P^m^ and β^m^ are the weight, latent variable and regression coefficient matrices of the PLS model for X^m^ and Y^m^ in the master instrument (with selected number of latent variables (LVs)), the projection of the master’s transfer samples X^m^_ct_ in the PLS model T^m^_ts_ and the projection of the slave’s transfer samples X^s^_ct_ in the PLS model T^s^_ts_ are given by:

T^m^_ts_ = X^m^_ct_ W^m^ ((P^m^)^T^ W^m^)^−1^ (8)

T’^s^_ts_ = X^s^_ct_ W^m^ ((P^m^)^T^ W^m^)^−1^ (9)

Assuming a linear relationship between the projection matrices:

T^s^_ts_ = T’^s^_ts_ M = T^m^_ts_ (10)

where M can be obtained by the ordinary least squares method:

M = ((T’sts)T T’sts)−1 (T’sts)T Tmts(11)

when M is obtained, a classification/regression method can be applied on the projected matrices T or from the PLS model already built in the master instrument:

Y^s^ = X^s^ β^m^ (12)

Since in this work we use PLSDA for a classification problem, we will call this method PLSDA-CT instead of PLSCT.

Correlation alignment (CORAL)—CORAL [84] is an unsupervised domain adaptation method coming from the machine learning field that attempts to minimize the differences in the data distributions between two domains (master and slave instruments in our case) by transferring the data structure of the target domain (slave).

Notating X^m^ and X^s^ subsets of unlabeled data from master and slave, respectively, having N_m_ and N_s_ number of features each (X matrix columns), the proposed transformation is:

C_m_ = (X^m^)^T^ X^m^ + λ I_Nm × Nm_ (13)

C_s_ = (X^s^)^T^ X^s^ + λ I_Ns × Ns_ (14)

X^m^ = X^m^ C_m_^−1/2^ C_s_^1/2^ (15)

where C^m^ and C^s^ are the master’s and slave’s data covariance matrices, respectively, adapted with a small regularization parameter λ that allows it to be full rank and thus the square root to be computed. Therefore, CORAL uses two steps to align both data distributions: whitening the master data and re-coloring it with the slave covariance.

### 3.2. Data Analysis Methodology

In this work, we study the performance of several CT algorithms to transfer calibration models between pairs of 3 instruments in an experiment consisting of an on-site real breath analysis study for discrimination of breath samples from subjects before and after eating a specific meal. Thus, data is classified into classes “meal” and “no-meal”. We use device 1 as the master device, thus devices 2 and 3 are considered the slave devices. Denoting the master instrument as M and the slave instrument as S, two instrument pairs considered are: M1–S2 and M1–S3.

To illustrate the effect of the different CT algorithms, we followed a procedure consisting of three steps. In the first step, dimensionality reduction and a classifier using data from M is built and evaluated with selection of their optimal parameters for each of the 3 devices. In the second step, a small number of samples (transfer samples) from both M and S are selected and the CT is performed. Finally, in the third step, the CT algorithm is evaluated using the classifier from the first step on S data. The result of the M classifier applied on the M data is considered the reference to be achieved, while the result of the M classifier applied on each S data is considered the threshold to be overcome.

Since only two classes are involved in the classification task, the classification results are given in area under the receiver operating characteristic curve (AUC) and standard errors (SE). Results for each S device are compared before and after the application of the CT algorithms; if the results of the corrected S data (after CT) are similar to the M’s reference classification result we consider a successful CT, if the S’s result is higher than the one before the CT (threshold) we consider good CT.

Classifier—First, the basic pre-processing step here is given by the ratio of the sensor’s conductance (1/R) with the baseline, which gives R0/R, where R0 is the sensor output resistance to room air (baseline) and R is the sensor’s response resistance to the breath exposure. The considered R is one value that summarizes the sensor’s response to the whole analyte exposure. It corresponds to the mean of the latest measurements before the cleaning step, in this case the last 5 s, when the sensor responses are most stable (steady state). Therefore, the resulting data sets from measurements with our 18 sensors have 18 columns, one per sensor.

As specified above, the results of the classifier built in M and applied on each S is considered the reference for the evaluation of the CTs. The considered classifier is linear discriminant analysis (LDA) with a previous dimensionality reduction task using principal component analysis (PCA). Therefore, the number of PCA components (nPCs) for the PCA + LDA classifier is the parameter to be selected. For this, cross-validation is made on two random subsets of the M’s data: a training set containing 48 samples with equally represented classes (24 + 24), and a validation set with the remaining samples (~39). The procedure is repeated 20 times for each parameter value to obtain the optimal nPC.

Transfer samples—Once the optimal nPCs for the classifier in M are selected, the CT task is carried out as follows. For every pair M–S, a sample subset (M-training set) is selected from M with both classes equally represented. The M’s transfer samples are selected from this subset. Then, the S’s transfer samples are selected according to the CT algorithm as described below, and the CT is performed.

The CT algorithms need a number of samples (transfer samples), labeled or not, from both M and S. To select the transfer samples from M, we use first the Kennard–Stone algorithm (KS) [88], and then for CT methods that use labeled transfer samples, the transfer samples from S are selected according to standard samples or nonstandard samples. Instead, for CT methods that use unlabeled transfer samples, KS is also used to select the S’s transfer samples. The standard samples correspond to the M’s equivalent samples in S, this is, the samples that were taken close in time (the subjects exhaled on each instrument right one after the other) from the same patients by the 3 instruments. In turn, the non-standard transfer samples are an alternative to the standard samples and correspond to the very likely case where standard samples as defined above are not available. Non-standard transfer samples are selected as follows: once M’s transfer set is selected with KS, from a subset of 20 known (labeled) S samples, each selected S transfer sample is the closest to each M’s transfer sample.

In addition, for cases with labeled samples the selection of M and S transfer samples is made according to 2 class membership conditions: samples belonging to (a) both classes in the experiment (meal/no-meal) or (b) only one class corresponding to no-meal. As for the healthy class in a disease-control breath analysis experiment, subjects belonging to no-meal are easier to collect and thus, a CT method based on only such sample class would be more practical.

**Figure 5:**
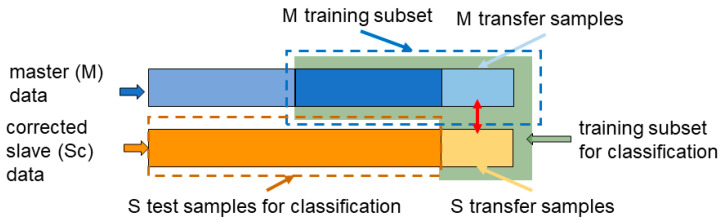
Visual scheme of training set for the calibration transfer evaluation.

CT evaluation—To assess the CT algorithms’ performance, the selected M classifier is built using the M’s training samples and the S’s transfer samples if they are labeled. The classifier is then applied on the corrected S’s samples excluding its transfer samples (Figure 5).

An additional reference can be considered for the CT algorithms that need known labeled transfer samples from S; the calibration extension method (CEM) which consists of the LDA classifier built with M’s training samples plus original (non-corrected by CT) S’s labeled transfer samples, this is S’s samples without CT. Then, the CT can be considered good if its AUC overcomes the CEM’s AUC. The procedure is repeated 20 times and the AUC and SE are computed.

The number of considered transfer samples is 10, 20, 30 and 40. If the transfer samples are labeled, as for DS, PDS and PLSDA-CT, the classes are equally represented within them.

## 4. Results and Discussion

### 4.1. The Dataset

After removing few outliers using PCA, the data set composition of the breath analysis experiment is shown in Table 1.

**Figure 6:**
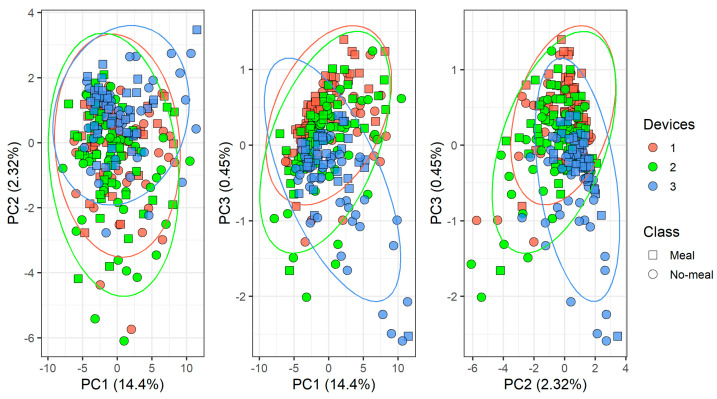
Principal component analysis (PCA) scores plot of the data according to the devices.

**Figure 7:**
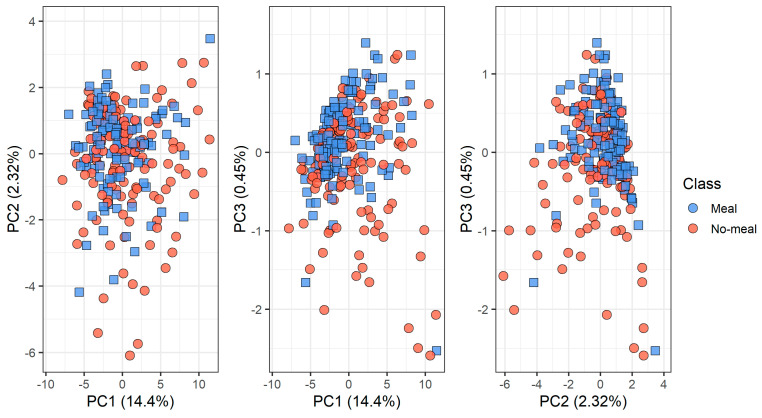
PCA scores plot of the data according to the classes: meal/no-meal.

Figure 6 and Figure 7 show the PCA scores plots of the complete data set according to the devices and the meal status (classes), respectively. Measurements from the three devices depend strongly on the device, since the breath samples come from the same individuals and the sampling is made on each device one right after the other (Figure 6). On the other hand, a certain degree of overlap between the data classes meal/no-meal can be seen in Figure 7. This happens in every device, as it is shown by the sample symbols shown in Figure 6.

### 4.2. Classification

The references to compare the performance of the CTs are the results of the classification of every device’s data using a PCA + LDA model built with M’s training set data (48 samples) before the CT. Table 2 shows the classification results for the PCA + LDA models giving the best AUC according to device master-slave pairs, which indicates: device to build model-device to test model. For example, for pairs with the same device as M1–M1, it indicates that a training set (~48 samples) from M1 was used to build the model and a test set (~39 samples) from the same device was used to evaluate it. When the pairs are formed by different devices, the number of samples in the training set is ~48 samples but for the test set is ~80 samples.

We can see in Table 2 that results for pairs of the same devices show good results for discriminating human breath before and after the meal for the individual devices. However, there is a significant performance decrease when the classifier is built with the master device, which is a clear indicator of the fact that the devices differ.

The selected reduced dimension obtained after cross-validation (13 PCs) corresponds to the model with optimal results in M1 (89.26 ± 0.87). The same model applied to devices 2 and 3 gave 73.15 ± 1.15 and 75.72 ± 2.40, respectively. These values are the lower value reference for the evaluation of the methods (shadowed in Table 2).

### 4.3. Calibration Transfer Using Two-Class Transfer Samples

**Figure 8:**
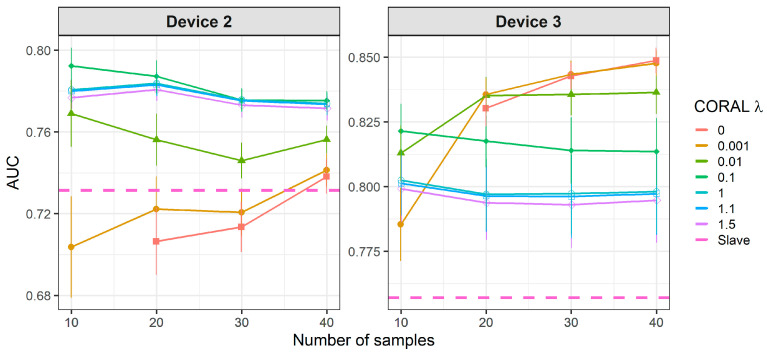
Classification results for correlation alignment (CORAL) using two-class standard transfer samples.

**Figure 9:**
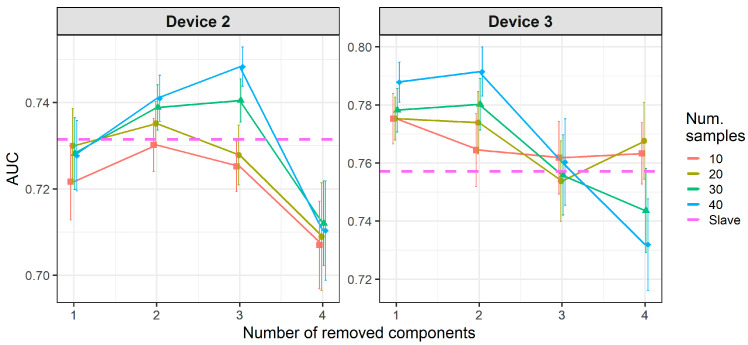
Classification results for partial least squares discriminant analysis (PLSDA) using two-class standard transfer samples.

**Figure 10:**
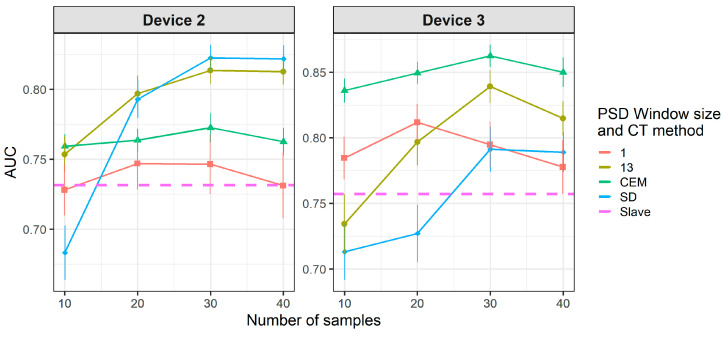
Classification results for CEM, DS and PDS using two-class standard transfer samples.

**Figure 11:**
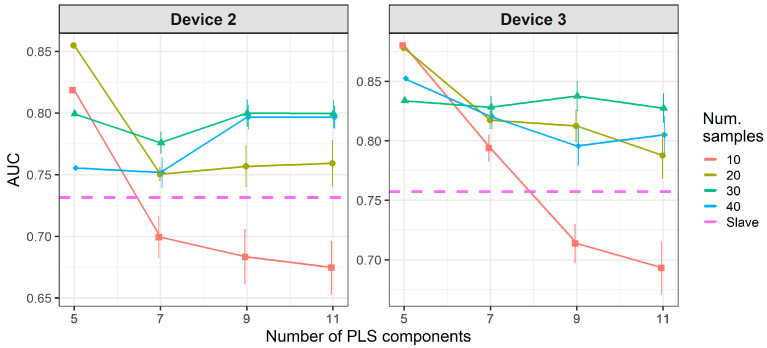
Classification results for using two-class standard transfer samples.

Figure 8 and Figure 9 show the results of CT methods CORAL and PLSDA for both slave devices. These are methods that do not need labeled samples from S. However, since results for CORAL depend on λ and PLSDA depend on nLV, some known samples in the slave device must be known in order to find an optimal value. Results for CORAL depend on the device but good results are obtained for both devices at high λ with low dependency on the number of transfer samples. AUC results for the low λ increase with the number of transfer samples. The lowest λ give the best results for device 3, while it is the contrary for device 2. In turn, for PLSDA the optimal number of PLSDA components to be removed depends on the slave device and the number of transfer samples. Best results are obtained with the maximum number of transfer samples. Figure 10 and Figure 11 show the results of CT methods DS, PDS and PLSDA-CT for both slave devices, when using standard samples. CEM is a reference which shows whether it is worth applying any of these CT methods or simply building the CEM classifier with labeled samples from each pair of M and S devices together.

Figure 10 shows that when using standard transfer samples, PDS can give good results in on-field experiments, although with many more transfer samples than those reported for lab experiments. The optimal parameters for PDS or DS depend on the slave device and number of transfer samples. For device 3, it is better to use CEM than the PDS as CT. For both slave devices, PDS at the maximum window size (13) and a high number of transfer samples gives the best results. In turn, when using PLSDA-CT (Figure 11) we can see that the best results are obtained with few components and medium number of transfer samples. However, good and more stable results with respect to the nLVs are obtained with many transfer samples.

**Figure 12:**
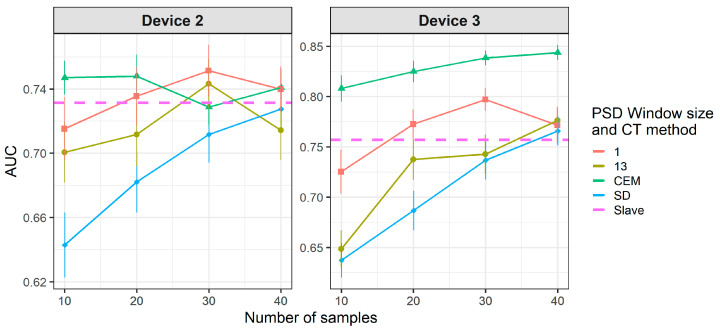
Classification results for CEM, DS and PDS using two-class non-standard transfer samples.

**Figure 13:**
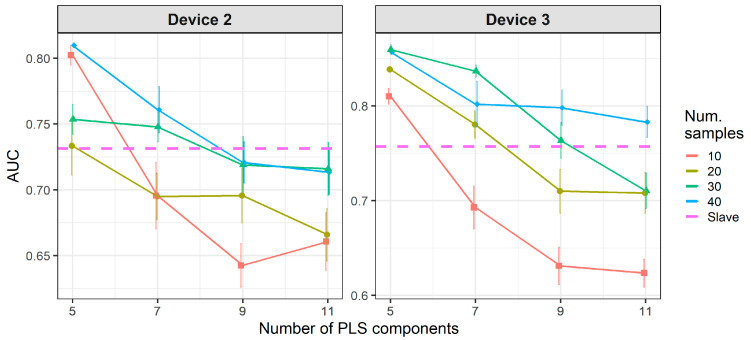
Classification results for PLSDA-CT using two-class non-standard transfer samples.

Figure 12 and Figure 13 show that there is a considerable decrease in the general performance of PDS, DS and PLSDA-CT methods when using non-standard transfer samples. CEM results, which are similar to the case of standard transfer samples above, are still the best for device 3 (as in Figure 10). However, for device 2 it becomes comparable to PDS with window size 1, while DS and PDS for window size 13 drop below the threshold. Window size equal to 1 for PDS does not give good results but it keeps the values mostly above the threshold level for both devices at the two cases of transfer samples used. Given the general behavior of DS in Figure 9 and Figure 10, for a study with more data for which more transfer samples were available (thus bigger training set size) much better rates could be achieved. The general worsening of the results can also be seen for PLSDA-CT. However, AUC is still good for the smallest number of LVs.

### 4.4. Calibration Transfer Using One-Class Transfer Samples

**Figure 14:**
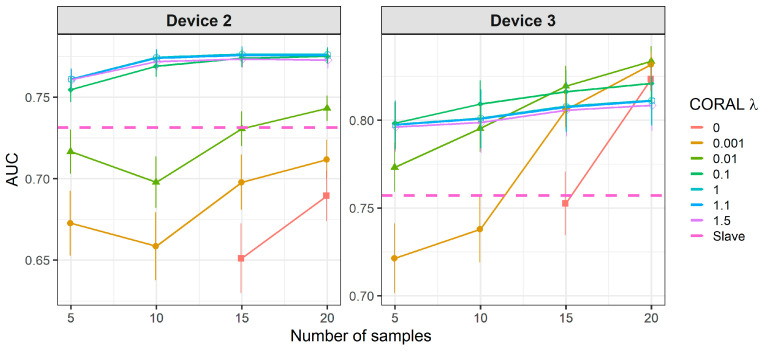
Classification results for CORAL using one-class transfer samples from class “no-meal”.

The following figures show results for the case of using transfer samples of only one class, “no-meal” in our case. Figure 14 shows results for CORAL, which behaves similarly although slightly worse than for the previous case (Figure 8). The high λ values give close results which are stable with respect to the number of transfer samples, while small λ values give increasingly better results with increasing number of transfer samples. In fact, best results for device 3 are given by the smallest λ but it is the contrary for device 2. Unfortunately, in this case none of the results given by PLSDA overcomes the threshold, therefore we do not show them here.

**Figure 15:**
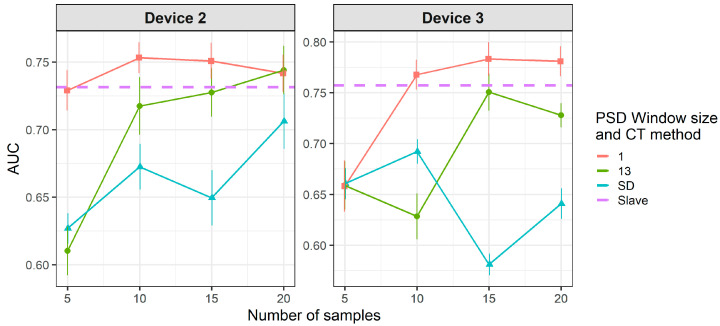
Classification results for DS and PDS using one-class standard transfer samples from class “no-meal”.

**Figure 16:**
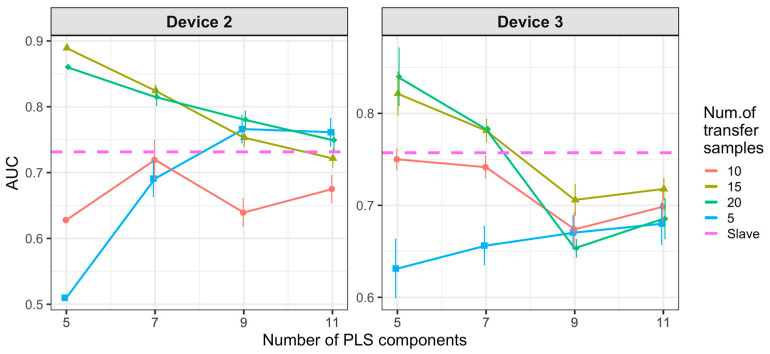
Classification results for PLSDA-CT using one-class standard transfer samples from class “no-meal”.

Results for DS and PDS are not good when using one-class standard transfer samples, only PDS with window size equal to 1 gives AUC slightly over the threshold (Figure 15). However, PLSDA-CT still shows good results for small numbers of LVs but only for high number of transfer samples (Figure 16).

**Figure 17:**
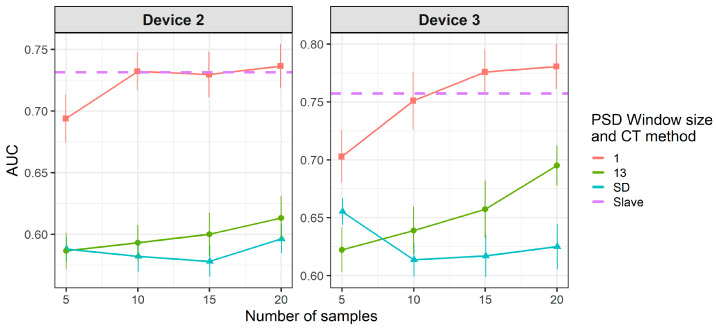
Classification results for DS and PDS using one-class non-standard transfer samples from class “no-meal”.

**Figure 18:**
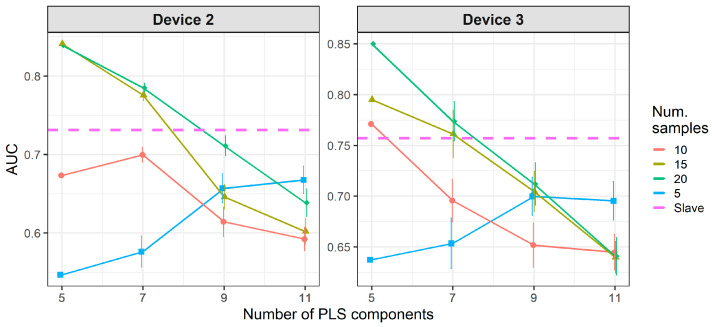
Classification results for PLSDA-CT using one-class non-standard transfer samples from class “no-meal”.

Finally, the use of one-class non-standard transfer samples results in Figure 17, which shows a worsening in the performance of DS and PDS and a similar behavior of PLSDA-CT. In addition, PLSDA-CT shows a change in the trend for the smallest number of one-class transfer samples (Figure 16 and Figure 18) of the performance, which increases with the LVs, with respect to two-class transfer samples (Figure 11 and Figure 13) for which the performance decreases with the LVs.

In summary, we have shown the performance of several CT methods using labeled transfer samples (DS, PDS, PLSDA-CT) with samples from one or two classes, and using unlabeled transfer samples (CORAL, PLSDA) knowing that they contain one or two classes. Since CORAL is based on covariance and in our data set the covariance of both classes are not dramatically different (so we can use LDA as classifier), its performance using a transfer sample set with one class is not much worse than using two classes. On the contrary, PLSDA needs both classes in the transfer sample set to find a component on a suitable direction to be removed. In turn, due to their nature, DS and PDS are very sensitive not only to the classes present in the transfer set but also to the type of samples. Therefore, standard samples of both classes are necessary for correct performance. Finally, results for PLSDA-CT are more robust and stable for standard samples of both classes, but for a small number of components and a high number of transfer samples, PLSDA-CT is giving good results for all cases.

## 5. Conclusions

In this work, we have presented a novel, compact and easy-to-use breath analyzer platform with a modular sensing chamber and direct breath sampling unit. Furthermore, we have tested the performance of four calibration transfer methods in a breath analysis experiment using real human breath measurements to classify breath samples of subjects before and after eating a specific meal. In our study the breath measurement is taken about 4 h after the food intake; this leads to the conclusion that the sensors are affected by every food intake. This can be viewed as a potential disorder in studies with healthy and sick people and should be considered when designing an appropriate sampling protocol.

The measurements were made using three instruments. One of them (device 1) was selected as the master instrument, so that its measurements were used to build classification models along with transfer samples whenever their classes were known. The other two instruments were the slave instruments.

The four CT methods tested follow very different approaches, especially with regard to the transfer samples they use. The test of these CT methods is in fact focused on the transfer samples they need for an acceptable performance as a response for a practical problem that arrives in on-field experiments, in our case in experiments using real human breath measurements with gas sensor-based instruments. In such experiments, measurements of samples at different locations and with different instruments are usual. The problem arrives when transferring the calibration from the master to the remaining slave instruments, because labeled samples from the slaves are needed and sometimes, they are difficult to obtain. We wondered firstly if real sample measurements (instead of lab-samples) could be used as transfer samples, and if so, how many and whether or not they must contain all classes under study in the classification problem.

In the figures above, we have shown that real human breath measurements can be used as transfer samples, although in large numbers, much larger than in lab experiments, and with results that depend on the device. However, we could derive some general conclusions. First, in all cases that need labeled transfer samples, the best performance of all methods was obtained for two-class standard samples, and a decrease could be seen when the two-class samples were not standard. Methods like PLSDA, DS and PDS need transfer samples containing all the classes involved in the classification problem, although for PLSDA these samples do not need to be specifically known. However, PLSDA-CT gives good results for small LVs and large transfer samples which in our experiment could contain only one class. CORAL also shows good results for both one and two-class unlabeled transfer samples, although it depends on the device and a parameter. Therefore, calibration transfer methods such as CORAL and PLSDA-CT could be used in on-field experiments using transfer samples from the samples under study, without the need of laboratory samples specifically measured for calibration transfer tasks or for recalibration purposes.

## Figures and Tables

**Table 1:** Data set composition.

Device	Meal	No-Meal
1	41	45
2	43	45
3	42	45

**Table 2:** Classification results before calibration transfer (CT).

Pair Train-Test Device	AUC (%)	Accuracy (%)	Sensitivity (%)	Specificity (%)	PCA nPCs
M1–M1	89.26 ± 0.87	80.01 ± 0.14	84.11 ± 0.20	75.75 ± 0.22	13
S2–S2	93.34 ± 0.65	86.41 ± 0.10	85.53 ± 0.17	87.25 ± 0.16	-
S3–S3	91.03 ± 0.12	81.56 ± 0.17	80.63 ± 0.30	82.50 ± 0.21	15
M1–S2	73.15 ± 1.15	64.83 ± 0.09	49.65 ± 0.26	79.66 ± 0.20	13
M1–S3	75.72 ± 2.40	66.88 ± 0.18	79.00 ± 0.38	54.75 ± 0.61	13

## Data Availability

The data presented in this study are available on request from the corresponding author. The original data are not publicly available due to privacy of participants.

## Supplementary Materials

The following are available online, Figure S1: Internal view with description of the three main units of the Modular Breath Analyzer (MBA) platform for laboratory and clinical use, Table S1: List of integrated analog sensors, number of obtained signals from each sensor and used heater voltages (adapted with permission from Jaeschke et al., Copyright 2018 by the authors), Table S2: List of integrated digital sensors, number of obtained signals from each sensor and used heater voltages (adapted with permission from Jaeschke et al., Copyright 2018 by the authors).
