# Supplementary material for: Modular Breath Analyzer (MBA): Introduction of a Breath Analyzer Platform Based on an Innovative and Unique, Modular eNose Concept for Breath Diagnostics and Utilization of Calibration Transfer Methods in Breath Analysis Studies
Source: Molecules. 2021 Jun 21;26(12):3776. doi: 10.3390/molecules26123776 (PMC8235513; doi:10.3390/molecules26123776)
Supplement: Supplementary file 1 [file molecules-26-03776-s001.zip › molecules-1246082-supplementary_revised version3.pdf]

Supplementary Materials

Modular Breath Analyzer (MBA): Introduction of a breath analyzer platform based on an innovative and unique, modular eNose concept for breath diagnostics and utilization of calibration transfer methods in breath analysis studies.

Authors: Carsten Jaeschke [1,†], Marta Padilla [2,†], Johannes Glöckler [1,†], Inese Polaka [3], Martins Leja [3], Viktors Veliks [3], Jan Mitrovics [2], Marcis Leja [3] and Boris Mizaikoff [1,*]

1 University of Ulm, Institute of Analytical and Bioanalytical Chemistry, Albert-Einstein-Allee 11, 89081 Ulm, Germany.
2 JLM Innovation GmbH, Vor dem Kreuzberg 17, 72070 Tuebingen, Germany.
3 Institute of Clinical and Preventive Medicine, University of Latvia, Riga, Latvia.
* Correspondence: boris.mizaikoff@uni-ulm.de; Tel.: +49-731-5022750
† These authors contributed equally.

Table of Content

Figure S1. Internal view with description of the three main units of the Modular Breath Analyzer (MBA) platform for laboratory and clinical use.

Table S1. List of integrated analog sensors, number of obtained signals from each sensor and used heater voltages (adapted with permission from Jaeschke et al. [1] Copyright 2018 by the authors).

Table S2. List of integrated digital sensors, number of obtained signals from each sensor and used heater voltages (adapted with permission from Jaeschke et al. [1] Copyright 2018 by the authors).

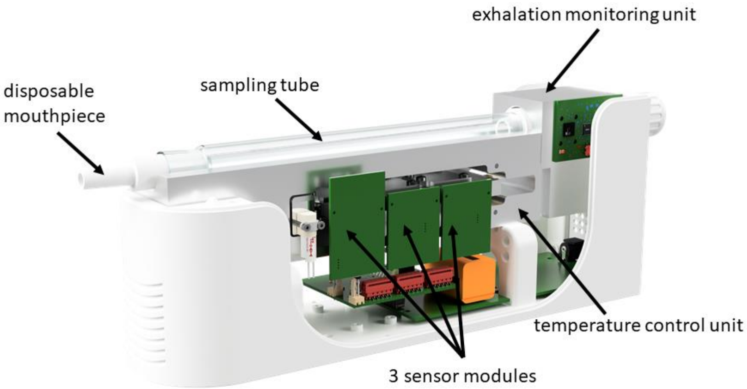

Figure S1. Internal view with description of the three main units of the Modular Breath Analyzer (MBA) platform for laboratory and clinical use.

Table S1. List of integrated analog sensors, number of obtained signals from each sensor and used heater voltages (adapted with permission from Jaeschke et al. [1] Copyright 2018 by the authors).

Sensor type	Nr. of incorporated sensors	Sensor output signals	Heater voltage [V]	References
MICS 6814	1	3	NH_3: 2.2<br>red: 2.4<br>ox: 1.7	[2]
MICS 4514	1	2	red: 2.4<br>ox: 1.7	[3]
TGS 8100	1	1	1.8	[4]
CCS 801	2	1	1.8	[5]

Table S2. List of integrated digital sensors, number of obtained signals from each sensor and used heater voltages (adapted with permission from Jaeschke et al. [1] Copyright 2018 by the authors).

Sensor type	Num of incorporated sensors	Sensor output signals	Applied supply voltage [V]	Applied temp mode	References
CCS 811	2	1	1.8	-	[6]
SGP30	2	2	1.8	-	[7]
ZMOD 4410	2	3*	1.8	Ramp and Flat	[8]
BME 680	2	4**	1.8	-	[9]

*controlled via Firmware (e.g. ramps), ** temperature, humidity, pressure and VOC

References

1. Jaeschke, C.; Gonzalez, O.; Glöckler, J.J.; Hagemann, L.T.; Richardson, K.E.; Adrover, F.; Padilla, M.; Mitrovics, J.; Mizaikoff, B. A Novel Modular eNose System Based on Commercial MOX Sensors to Detect Low Concentrations of VOCs for Breath Gas Analysis. In Proceedings of the Multidisciplinary Digital Publishing Institute Proceedings; **2018**; Vol. 2, p. 993.

2. SGX Datasheet MiCS-6814 Available online: https://www.sgxsensortech.com/content/uploads/2015/02/1143_Datasheet-MiCS-6814-rev-8.pdf (accessed on May 4, 2021).

3. SGX Datasheet MiCS-4514 Available online: https://www.sgxsensortech.com/content/uploads/2014/08/0278_Datasheet-MiCS-4514.pdf (accessed on May 4, 2021).

4. Figaro Datasheet TGS8100 Available online: https://www.figaro.co.jp/en/product/docs/tgs8100_product infomation(en)_rev06.pdf (accessed on May 4, 2021).

5. AMS Datasheet CCS 801 Available online: https://ams.com/documents/20143/36005/CCS801_DS000457_3-00.pdf/59f95f8c-2db9-91f8-c696-0056aa4cca55 (accessed on May 4, 2021).

6. AMS Datasheet CCS 811 Available online: https://ams.com/documents/20143/36005/CCS811_DS000459_7-00.pdf/3cfdaea5-b602-fe28-1a14-18776b61a35a (accessed on May 4, 2021).

7. Sensirion Datasheet SGP30 Available online: https://www.sensirion.com/fileadmin/user_upload/customers/sensirion/Dokumente/0_Datasheets/Gas/Sensirion_Gas_Sensors_SGP30_Datasheet.pdf (accessed on May 4, 2021).

8. IDT Datasheet ZMOD4410 Available online: https://www.idt.com/document/dst/zmod4410-datasheet (accessed on May 4, 2021).

9. Bosch Datasheet BME680 Available online: https://ae-bst.resource.bosch.com/media/_tech/media/datasheets/BST-BME680-DS001-00.pdf (accessed on May 4, 2021).
